# A Call for Screening: Hearing Loss and Chronic Otitis Media in Immigrants to Sweden

**DOI:** 10.1002/lio2.70397

**Published:** 2026-04-01

**Authors:** Sofie Kraft, Ylva Dahlin‐Redfors, Radoslava Jönsson, Nina Pauli

**Affiliations:** ^1^ Department of Otorhinolaryngology Region Västra Götaland, Sahlgrenska University Hospital Gothenburg Sweden; ^2^ Department of Otorhinolaryngology, Head and Neck Surgery, The Sahlgrenska Academy, Institution of Clinical Sciences University of Gothenburg Gothenburg Sweden

**Keywords:** chronic otitis media, hearing loss, immigration, otology

## Abstract

**Background:**

The prevalence of hearing loss and chronic otitis media (COM) varies globally and is reported to be higher in developing countries. Hearing loss and COM may hinder language learning after immigration and thus impede integration into society. The aim of the study was to evaluate the prevalence of hearing loss and COM in immigrants to Sweden.

**Material and Methods:**

Prospectively collected data regarding the hearing and general health of immigrants attending Swedish language classes were assessed. Participants with hearing loss were followed up with microscopic ear examination, pure tone audiometry including tympanometry, and questions regarding general health and health‐related quality of life.

**Result:**

In total, 487 participants were included. The mean age was 38.7 years, and the female to male ratio was 3 to 1. The most common countries of origin were Syria (16%), Somalia (13%), and Iraq (10%). Hearing loss was found in 74 of the participants (15.2%) and COM in 11 subjects (2.3%). Participants originating from Somalia had a significantly increased risk of COM, odds ratio (OR) 5.83 (95% CI 1.44–23.63), *p* = 0.036.

**Conclusion:**

We found that the prevalence of COM in the study cohort was 2.3% and hearing impairment in adult immigrants to Sweden attending Swedish language classes was 15.2%. Among the male participants, hearing loss was present in 28%. Over 25% of the participants with hearing loss were referred for hearing aid fitting. As learning a new language is crucial to integration into society, we advocate for the inclusion of screening audiometry in the general health check‐up for immigrants.

**Level of Evidence:**

3.

## Graphical abstract

The prevalence of chronic otitis media was 2.3% and hearing impairment was found in 15.2% in this cohort of adult immigrants to Sweden attending Swedish language classes. As learning a new language is crucial to integration into society, we advocate for the inclusion of screening audiometry in the general health check‐up for immigrants.

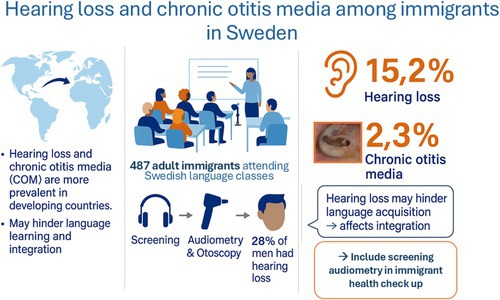

## 1 Introduction

Globally, hearing impairment is common, especially in developing countries [1]. The World Health Organization (WHO) estimates that around 1.5 billion people experience some degree of decline in their hearing capacity, around 430 millions of whom have hearing impairment requiring healthcare [2]. The etiology of hearing loss is multi‐factorial and includes increased age and hereditary and environmental factors, such as access to healthcare for treating infections and preventive measures against harmful noise exposure [3].

Chronic otitis media (COM) encompasses a broad range of conditions involving the middle ear and tympanic membrane. COM can be associated with inflammation of the middle ear mucosa, so called suppurative COM, and can cause hearing loss [4]. Acute otitis media (AOM) is a common infection of the middle ear and may cause a permanent ear drum perforation. In some cases, COM is associated with cholesteatoma, a mass lesion consisting of stratified keratinizing squamous epithelium, which can also lead to hearing loss and more serious complications such as facial palsy, intracranial abscess, and balance problems [5].

The incidence of COM in Western populations has been estimated to be around 9–17:100,000 per year [5, 6]. The condition is more common in developing countries and some indigenous groups [7, 8]. Risk factors associated with COM include smoking and low socioeconomic status, as well as recurrent upper respiratory infections [9]. For the purpose of this study, we defined COM as a perforation that persists for at least 12 weeks [10].

The hearing loss associated with COM is mainly conductive in nature, although it has also been proposed that COM can cause sensorineural hearing loss over time [11]. The degree of hearing loss may be partially improved with surgery [12].

The overall societal impact of hearing loss is substantial, due to reduced income for affected individuals, direct medical costs, and governmental compensation costs [13]. It has also been shown that adults with hearing loss have higher rates of unemployment and lower income later in life [14]. Moreover, people with hearing loss experience higher levels of social isolation, anxiety, and depression [15, 16].

While hearing impairment among immigrant populations has been addressed in previous research, particularly in pediatric and retrospective studies [17, 18], prospective data focusing on the prevalence of hearing impairment and COM in adult immigrant populations remain limited. The aim of this study was to investigate the prevalence of hearing impairment and COM in immigrants to Sweden attending Swedish language education classes. Furthermore, we aimed to evaluate health‐related quality of life and general health in the group of participants with hearing loss.

## 2 Materials and Methods

### 2.1 Study Design

The study was based on prospectively collected data regarding hearing function in immigrants attending Swedish language education classes for immigrants (Svenska för invandrare, SFI) in Gothenburg. Inclusion criteria were age over 18 years, being a legal immigrant to Sweden, and currently enrolled as a student studying Swedish at SFI classes. The ability to understand and give informed consent to the study was also required. The study information and questionnaire were available in eight languages, translated by a professional language translation service. Furthermore, a language teacher was available for assistance with translation if necessary.

Audiometry screening was offered to immigrants attending municipal adult language education classes, together with a study‐specific questionnaire. Results from the screening audiometry have previously been reported by the research group [19].

**FIGURE 1:**
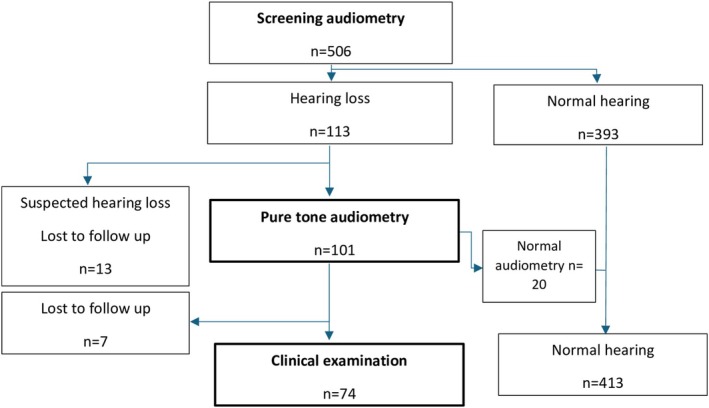
Study flowchart.

Participants who did not pass screening audiometry were included for further assessment with pure tone audiometry at a specialist Ear, Nose, and Throat (ENT) clinic. Participants with confirmed hearing loss underwent further evaluation by an ENT specialist; see the study flowchart in Figure 1.

Participants with initial screening audiometry pure‐tone average (PTA^4^) of more than 25 dB hearing level in any ear or > 40 dB HL on any single frequency were offered pure‐tone audiometry with threshold determination. The audiometry was carried out by an audiologist at an ENT clinic for frequencies 250, 500, 1000, 2000, 3000, 4000, 6000, and 8000 Hz (air conduction) up to a maximum presentation level of 90 dB. The PTA^4^ was calculated as the mean of frequencies 500, 1000, 2000, and 4000 Hz. The high frequency average (HPTA^3^) was calculated as a mean from 4000, 6000, and 8000 Hz. Bone conduction thresholds were tested for frequencies 500, 1000, 2000, and 4000 Hz.

Hearing impairment was defined as PTA^4^ > 25 dB HL in any ear or > 40 dB HL on a single frequency. High‐frequency hearing loss was defined as HPTA^3^ > 25 dB HL. Grading of hearing loss was in accordance with the 2019 WHO definition as follows: mild hearing loss 26–40 dB, moderate 41–60 dB, and severe > 60 dB [20, 21].

### 2.2 Clinical Examination

Subjects with hearing impairment were examined by an ENT specialist with regards to eardrum status using a microscope. The examination included documentation of ear status with otoscopic footage and assessment of hearing‐related medical history, including exposure to harmful noise, hereditary factors, and prior ear disease.

### 2.3 Study Questionnaires

The screened participants were assessed with a study‐specific questionnaire including questions regarding country of origin, years spent in Sweden, education level, sex, and age, as well as questions from the Swedish National Health Survey regarding general health, smoking status, and comorbidities, that is, asthma, diabetes, hypertension, and allergies.

Participants with hearing impairment also completed the EuroQoL 5‐dimensions 3‐level, EQ‐5D‐3L instrument [22]. This questionnaire addresses general perceived health with five questions regarding mobility, self‐care, usual activities, pain, and anxiety, as well as quality of life estimates using a Visual Analog Scale (VAS). The validated Swedish‐language version of the questionnaire was used, and all participants received help from an interpreter in their native language as needed.

### 2.4 Statistical Methods

For correlation analyses, Fisher's exact test was used to determine whether significant differences existed between study participants with and without hearing loss. All tests were two‐tailed and performed at the 5% significance level, with a *p* value of < 0.05 considered significant. Univariate logistic regression analysis was carried out, and variables with a significance value of *p* < 0.05 were further analyzed with multivariate logistic regression analysis. Furthermore, risk factors for COM were analyzed with univariate logistic regression analysis.

### 2.5 Ethics

This study was approved by the Ethical Review Board in Sweden (dnr 2019‐023589) and performed in accordance with the Declaration of Helsinki. All participants gave their written informed consent to participate.

## 3 Results

### 3.1 Study Participants

A total of 506 immigrants attending Swedish language classes were evaluated with audiometric screening. One hundred and thirteen (22%) did not pass the initial audiometric screening. After pure tone audiograms were carried out, 20 had normal hearing levels. Nineteen were lost to follow‐up and therefore excluded. In total, a study cohort of 487 participants was available for analysis: 74 (15%) with confirmed hearing loss and 413 (85%) with normal hearing, see Figure 1.

### 3.2 Study Group Characteristics

The majority of the study cohort were women, with a female to male ratio of about 3:1, as shown in Table 1. In the group of male participants, there was a significantly higher proportion with hearing loss 28% (33/118), compared to 11% (41/369) among female participants. The overall mean age was 38 years, but in the group with hearing loss, it was higher, 46.4 years. The mean time spent in Sweden was 4.2 years. Approximately 60% had completed elementary school as their highest academic level. The most common regions of origin were Asia and the Middle East, and the most frequently represented countries were Syria, Somalia, Iraq, and Afghanistan.

**TABLE 1:** Characteristics and comorbidities of study participants, with and without hearing impairment.

	Study population (*n* = 487)	Hearing loss (*n* = 74)	Normal hearing (*n* = 413)	Hearing loss vs. no hearing loss^a^
Age (years)				
Mean (SD)	38.6 (10.5)	46.4 (11.4)	37.2 (9.6)	
Years in Sweden (years)^b^				> 0.30
Mean (SD)	4.2 (3.7)	3.8 (2.9)	4.2 (3.8)	
Sex, *n* (%)				***p* < 0.001**
Male	118 (24.2)	33 (44.6)	85 (20.6)	
Female	369 (75.8)	41 (55.4)	328 (79.4)	
Education level, *n* (%)^b^				
Elementary school	295 (60.6)	48 (64.9)	247 (59.8)	*p* > 0.30
High school	93 (19.1)	14 (18.9)	79 (19.1)	
University/college	91 (18.7)	11 (14.9)	80 (19.4)	
Missing	8 (1.6)	1 (1.4)	7 (1.7)	
Country of origin *n* (%)				
Syria	80 (16.4)	13 (17.6)	67 (16.2)	> 0.30
Somalia	66 (13.6)	13 (17.6)	53 (12.8)	
Iraq	46 (9.4)	3 (4.1)	43 (10.4)	
Afghanistan	42 (8.6)	9 (12.2)	33 (8.0)	
Eritrea	29 (6.0)	2 (2.7)	27 (6.5)	
Iran	20 (4.1)	3 (4.1)	17 (4.1)	
Other	198 (40.7)	30 (40.5)	168 (40.7)	
Missing	6 (1.2)	1 (1.4)	5 (1.2)	
Geographic area of origin, *n* (%)				
Europe	43 (8.8)	6 (8.1)	37 (9.0)	*p* > 0.30
Asia	281 (57.7)	43 (58.1)	238 (57.6)	
Middle East	136 (27.9)	21 (28.4)	115 (27.8)	
Africa	7 (1.4)	1 (1.4)	6 (1.5)	
North America	0 (0.0)	0 (0.0)	0 (0.0)	
South America	14 (2.9)	2 (2.7)	12 (2.9)	
Missing	6 (1.2)	1 (1.4)	5 (1.2)	
Comorbidities, *n* (%)				
Asthma	41 (8.4)	7 (9.5)	34 (5.8)	> 0.30
Hypertension	52 (10.7)	13 (17.6)	39 (9.4)	0.072
Diabetes	27 (5.5)	9 (12.2)	18 (4.4)	**0.025**
Allergies	105 (21.6)	14 (18.9)	91 (22.0)	> 0.30
Missing	8 (1.6)	1 (1.4)	7 (1.7)	
General health, *n* (%)				***p* < 0.001**
Excellent	140 (29.4)	9 (12.3)	131 (32.4)	
Good	217 (45.5)	34 (46.6)	183 (45.3)	
Neutral	89 (18.7)	20 (27.4)	69 (17.1)	
Poor	26 (5.5)	9 (12.2)	17 (4.1)	
Very poor	5 (1.0)	1 (1.4)	4 (1.0)	
Missing	10 (2.1)	1 (1.4)	9 (2.2)	
Smoking, *n* (%)				
Yes	63 (13.3)	14 (19.2)	49 (12.2)	*p* = 0.16
Missing	13 (2.7)	1 (1.4)	12 (2.9)	

*Note:* Bold text indicates *p* > 0.05.

^a^ Fishers exact test, chi^2^ test, and Fisher's permutation test.

^b^ Missing *n* = 8, *n* = 1 hearing loss, and *n* = 7 normal hearing.

### 3.3 Hearing Outcome and Audiometric Result

**FIGURE 2:**
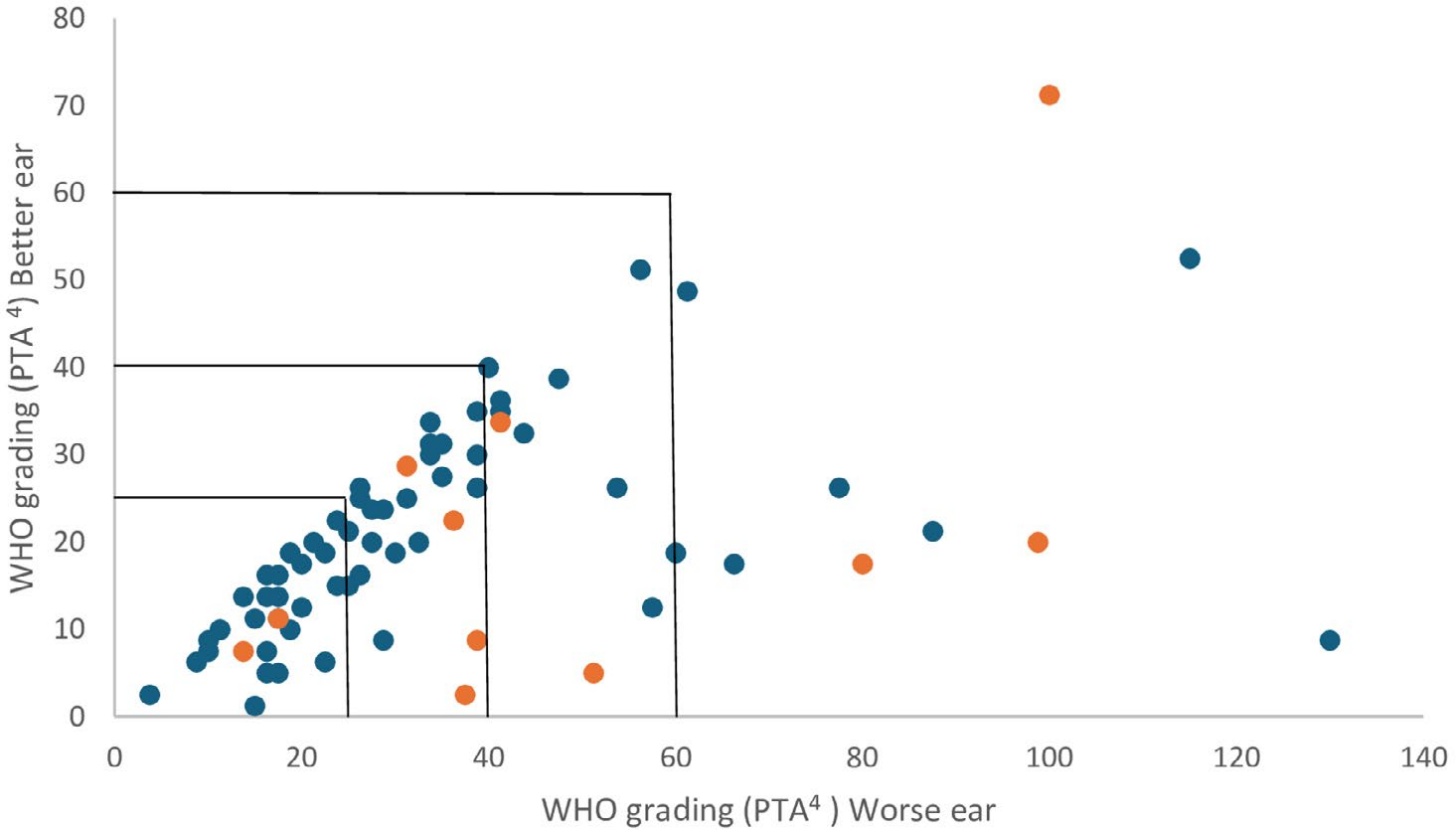
PTA^4^ for the better and worse ear in study participants with hearing impairment (*n* = 74). Orange color marks participants with chronic otitis media (*n* = 11).

We found that 74 of the 487 study participants (15.2%) had a hearing impairment according to the study criteria (PTA^4^ > 25 dB HL in any ear or > 40 dB HL on a single frequency), as shown in Table 2. The median values for PTA^4^ in the study group with hearing impairment were 19 dB HL in the better ear and 29 dB HL in the worse ear. The degree of hearing loss at the higher frequencies was more pronounced, as demonstrated by a median value on HPTA^3^ of 38 dB HL in the better ear and 50 dB HL in the worse ear. Nineteen study participants (25.7%) had moderate or severe hearing loss in their worse ear; four of these had bilateral hearing loss with a PTA^4^ > 40 dB. The prevalence of severe bilateral hearing loss was 1.4%. Thirty‐one participants (42.9%) had hearing loss of > 40 dB HL on a single frequency, see Figure 2.

**TABLE 2:** Pure‐tone audiometry in the group with hearing impairment, *n* = 74 (median dB 10–90 percentiles).

Frequency (Hz)	250	500	1000	2000	4000	6000	8000	PTA^4^ ^a^	HPTA^3^ ^b^
Better ear									
Air conduction (dB HL) (*n* = 74)	15 (5–40)	10 (0–35)	10 (0–35)	20 (0–30)	30 (5–55)	40 (15–65)	45 (15–70)	18.8 (6.3–35.0)	38.3 (16.7–63.3)
Bone conduction (dB HL) (*n* = 56)	—	15 (5–35)	10 (0–30)	20 (10–40)	20 (0–45)	—	—	—	—
Worse ear									
Air conduction (dB HL) (*n* = 74)	25 (5–65)	25 (5–65)	25 (5–60)	25 (5–70)	45 (20–75)	50 (50–105)	55 (30–105)	28.8 (13.8–66.3)	50.0 (30.0–95.0)
Bone conduction (dB HL) (*n* = 69)	—	15 (0–40)	15 (0–40)	30 (10–60)	30 (5–60)				

Abbreviations: HPTA^3^, high frequency pure‐tone average; PTA^4^, pure‐tone average.

^a^ Mean value for frequencies 500, 1000, 2000, and 4000 Hz.

^b^ Mean value for frequencies 4000, 6000, and 8000 Hz.

### 3.4 Hearing Loss Etiology

The prevalence of COM, defined as a persistent ear‐drum perforation, was 2.3% (*n* = 11 of 487) in the study cohort. Of the participants with hearing loss, 14.9% had COM with or without secretion, see Table 3. Immigrants originating from Somalia had an increased risk for COM (odds ratio [OR] 5.83, 95% CI 1.44–23.63, *p* = 0.036). Hearing results for participants with and without COM are shown in Figure 2.

**TABLE 3:** Hearing‐loss etiology, grading of hearing loss and medical recommendations in study participants with hearing impairment (total *n* = 74 if no other number is given).

Hearing loss etiology *n* (%)	
Chronic otitis media, with or without cholesteatoma	11 (14.9)
Otosclerosis	4 (5.4)
Noise‐induced hearing loss	11 (14.9)
Others	48 (64.9)
Hearing loss grading *n* (%)	
Worse ear	
Normal (PTA^4^ 0–25 dB)	31 (41.9)
Mild (PTA^4^ 26–40 dB)	24 (32.4)
Moderate (PTA^4^ 41–60 dB)	10 (13.5)
Severe (PTA^4^ > 60 dB)	9 (12.2)
Better ear	
Normal (PTA^4^ 0–25 dB)	51 (68.9)
Mild (PTA^4^ 26–40 dB)	19 (25.7)
Moderate (PTA^4^ 41–60 dB)	3 (4.1)
Severe (PTA^4^ > 60 dB)	1 (1.4)
High frequency hearing loss *n* (%)^a^	
HPTA^3^ > 25 dB, unilateral	18 (25.7) (mean 46 (SD 23.1))
HPTA^3^ > 25 dB, bilateral	53 (71.6) (mean 54 (SD 17.6))
Normal high frequency hearing *n* (%)	3 (2.7) (mean 19.5)
Medical recommendation, *n* (%)^b^	
Referral for hearing‐aid fitting	20 (27.0)
Medical treatment, e.g., topical drops	16 (21.6)
Surgical referral, e.g., tympanoplasty	3 (4.0)
Further medical investigations needed, e.g., MRI	19 (25.8)
Noise exposure *n* (%)	
Acoustical trauma, e.g., explosions, gunshots	12 (16.2)
Long term exposure to harmful noise	20 (27.0)
Missing	19 (25.7)

^a^ Mean value for frequencies 4, 6 and 8 kHz presented as *n*(%) and mean decibel value (SD).

^b^ More than one (1) alternative possible.

Of those with hearing loss (*n* = 74), four participants (5.4%) were diagnosed with otosclerosis based on clinical examination and findings on the audiogram, including stapedial reflexes, a prevalence of 0.8% in the study cohort. Twelve participants (14.9%) had possible noise‐induced hearing loss, with a positive medical history of either loud acoustic trauma or long‐term exposure to harmful noise levels, and after evaluation of audiograms showing either a classic dip on 4 kHz or sensorineural hearing loss not conclusive with other diagnoses, such as presbycusis.

### 3.5 General Health and Comorbidities

There was a statistically significant difference (*p* < 0.001) with lower perceived general health estimates in the group with hearing loss, see Table 1. In the group with normal hearing, most participants rated their general health as excellent (32.4%) or good (45.3%) compared to 12.3% and 46.6% in the group with hearing loss. In the group with hearing impairment, 12.2% had diabetes, compared with 4.4% in the group with normal hearing, *p* = 0.025. There was no statistically significant difference in the prevalence of asthma (8.4%), hypertension (10.7%), or allergies (21.6%) between the participants with normal hearing and those with hearing impairment. Of the study population, 13.3% were smokers, with no significant difference between the two groups.

### 3.6 Regression Analysis for Hearing Impairment

Univariate logistical regression analysis revealed statistically significant risks for hearing loss for male sex (*p* < 0.001), older age (*p* < 0.001), poor estimated overall health (*p* < 0.001), diabetes (*p* = 0.049), hypertension (*p* = 0.019), and tinnitus (*p* = 0.001).

Significant predictors (*p* < 0.05) were included in a multivariate logistic regression analysis. Male sex (OR 2.83, 95% CI 1.58–5.06), increased age (OR 1.08, 95% CI 1.05–1.11), and tinnitus (OR 1.93, 95% CI 1.34–2.79) were the variables with the strongest statistical relationship to hearing loss.

### 3.7 Medical Intervention

Nineteen participants with hearing loss (26%) were referred for in‐depth medical examination with, for example, magnetic resonance imaging (MRI), computed tomography (CT), or acoustic reflex testing to rule out vestibular schwannoma, cholesteatoma, and otosclerosis. Sixteen participants (22%) were prescribed topical ear drops for ongoing infection or inflammation. After further diagnostic testing, three participants were referred for surgery, either tympanoplasty or mastoidectomy for cholesteatoma. Four participants were diagnosed with otosclerosis with a significant air‐bone gap and were offered surgical treatment. Twenty participants (27%) were referred for hearing aid fitting, while three had been fitted with hearing aids prior to the study, see Table 3.

### 3.8 EQ‐5D‐3L

The quality of life of participants with hearing impairment was evaluated with the EQ‐5D‐3L. On the question regarding pain/discomfort 44.6% stated that they experienced moderate pain/discomfort, and 13.5% stated that they experienced extreme pain/discomfort. On the question regarding anxiety/depression 39.2% reported that they experienced moderate anxiety or depression, and 18.9% stated that they experienced extreme anxiety or depression. Regarding self‐care, mobility, and usual activities, reported impairments were uncommon, see Table 4.

**TABLE 4:** General health and quality of life of study participants with hearing impairment using the EQ‐5D‐3L, *n* = 74.

Mobility, *n* (%)	
I have no problems in walking about	62 (83.7)
I have moderate problems in walking about	12 (16.2)
I am confined to bed	0 (0.0)
Self‐care, *n* (%)	
I have no problems with self‐care	74 (100.0)
I have some problems washing or dressing myself	0 (0.0)
I am unable to wash or dress myself	0 (0.0)
Usual activities, *n* (%)	
I have no problems with performing my usual activities	66 (89.2)
I have some problems with performing my usual activities	8 (10.8)
I am unable to perform my usual activities	0
Pain/discomfort, *n* (%)	
I have no pain or discomfort	31 (41.9)
I have moderate pain or discomfort	33 (44.6)
I have extreme pain or discomfort	10 (13.5)
Anxiety/depression, *n* (%)	
I am not anxious or depressed	31 (41.9)
I am moderately anxious or depressed	29 (39.2)
I am extremely anxious or depressed	14 (18.9)
EQ VAS overall health rated status, 0–100 mean value (SD) (*median*)^a^	73.6 (23.7) (*80*)

^a^ An EQ VAS score of 100 is equivalent to the best possible overall health, and 0 to the worst possible overall health.

## 4 Discussion

We found that the prevalence of COM was 2.3% in this study of adult immigrants to Sweden attending Swedish language classes (SFI), and that the prevalence of hearing impairment was 15% in the study cohort. Of the study participants with hearing impairment, 27% were referred for hearing aid fitting after our evaluation. Only three participants had hearing aids fitted previously.

The demographics of our study cohort of SFI students correlate well with those of the general population of immigrants to Sweden regarding country of origin, with almost half of the participants, 48%, originating from Syria, Somalia, Iraq, or Afghanistan. The vast majority of participants were women, almost 75%, which is not representative of the slight male predominance seen among immigrants to Sweden, 52%, in the last two decades [23]. The ratio is, however, representative of that of daytime students attending SFI, where a possible explanation is that men, to a higher degree, tend to be evening students and are more likely to be working during daytime hours. This could lead to an underestimation of the true number of individuals with hearing loss in the whole population since males have been shown to have a higher prevalence of hearing loss [24]. There was a significantly higher proportion of men with hearing loss compared to women in this study.

In this study cohort, we found a prevalence of hearing loss of 15.2%, defined as PTA^4^ > 25 dB HL in any ear, or > 40 dB HL on a single frequency. Among male participants the prevalence was 28%. The estimated prevalence of hearing loss in the adult population is around 12.7%, according to large, national surveys in the United States, with hearing loss defined as hearing threshold > 25 dB HL in the worse ear [25]. The mean age of our study cohort was 38.6 years, but in the group with hearing loss, the mean age was higher, 46.4 years. The multivariate regression analysis showed a strong link between older age and increased risk of hearing loss, with an OR of 1.08 (95% CI 1.05–1.11). This is a well‐known association, and the estimated prevalence of hearing loss, defined as > 25 dB HL in the worse ear rises across age groups: 6% among 35–44 year olds, 11% among 45–54 year olds, and 25% among 55–65 year olds [26]. To compare with the general population in Sweden, we have earlier studied perceived hearing problems and found it to be 1.6 times more common among all immigrants and twice as common for immigrants above the age of 44 [19].

Hearing loss is a global health problem, and several studies have shown correlations between hearing loss and poorer academic results, higher rates of unemployment, and lower income [14, 27, 28]. In some studies, even a slight or unilateral hearing loss has been shown to adversely affect school grades in children and adolescents compared to those of normal hearing [29]. Moreover, those with hearing loss score lower on cognitive tests when there is disruptive background noise, and speech discrimination decreases more than would be expected in individuals with a sensorineural hearing loss [30, 31]. In four cities in Sweden, there are SFI classes for those with known hearing loss, but as shown in this study, many students at regular SFI classes have undiagnosed hearing impairment [32].

The link between depression and social withdrawal in those with hearing loss has also been more closely studied in the last few decades, and it has been shown that individuals with hearing loss score higher on depression scales than matched individuals with normal hearing [16]. In our study, we found that fewer participants in the group with hearing loss rated their general health as “excellent” or “good.” Regarding anxiety and depression, as many as 58.1% among participants with hearing loss stated that they experienced moderate or severe anxiety/depression on the EQ‐5D‐3L instrument. In comparison, in a cross‐country analysis of norm data for EQ‐5D‐3L, only 26.4% of the Swedish population reported anxiety/depression [33]. Although other factors could be at play, it has been shown that mental health and communication difficulties are key issues for resettlement [34]. In the same cross‐country population study, the norm data from Sweden revealed that 42.5% experienced pain on some level, ranging from mild to severe, and among our study participants this number was 58.1%.

It is well known that the incidence and prevalence of COM are higher in developing countries. According to the WHO, the estimated incidence of COM is 30 million cases each year globally [2]. The prevalence in high‐income countries is < 1%, while it ranges between 3% and 6% in Sub‐Saharan Africa and the South Pacific Islands [35]. The prevalence of hearing loss associated with otitis media is estimated to be around 200 million, affecting 3:1000 people [36]. Our study confirmed that the COM prevalence was 2.3% in the group of immigrants to Sweden, and that the odds for COM increased five‐fold in participants originating from Somalia, a country where access to healthcare is more limited. Since COM can cause hearing loss that can be remedied to some extent with medical and surgical intervention, it is important to identify groups with a higher prevalence [12, 37]. Four participants (5.4%) were diagnosed with otosclerosis, a prevalence of 0.8% in the study cohort, which is in line with an estimated prevalence of around 0.1%–2.1% in other studies [38, 39].

### 4.1 Study Strengths and Limitations

The strength of the study lies in the prospective collection of data in a comparatively large cohort of presumed healthy individuals. Other strengths were that the study participants were carefully evaluated by the same ENT specialist and that health history, ear‐related problems, psychological problems, and ear status were documented. A limitation of the study was the difficulty of assessing a non‐response rate, since the school from which the participants were recruited operated on a weekly admission basis. Also, the SARS‐CoV‐2 pandemic necessitated a pause in the collection of data. Another limitation was the high female‐to‐male ratio, which could lead to an underestimation of hearing loss and perhaps an overestimation of otosclerosis.

## 5 Conclusion

We found that the prevalence of COM in the study cohort was 2.3% and hearing impairment in adult immigrants to Sweden attending Swedish language classes was 15.2%. Among the male participants, hearing loss was present in 28%. Only a few of the study participants with hearing loss had previously been tested with audiometry, and only three had obtained hearing aids before the study. As learning a new language is crucial to integration into society, we advocate for the inclusion of screening audiometry in the general health check‐up for immigrants.

## Funding

This work was supported by the Rune och Ulla Amlövs Foundation, Swedish Medical Society, and Swedish Hearing Research Foundation.

## Conflicts of Interest

The authors declare no conflicts of interest.

## Data Availability

The data that support the findings of this study are available on request from the corresponding author. The data are not publicly available due to privacy or ethical restrictions.
